# The application of metagenomic next-generation sequencing in patients with infection or colonization caused by *Lichtheimia* species

**DOI:** 10.3389/fcimb.2023.1103626

**Published:** 2023-03-28

**Authors:** Lihua Chen, Weiting Peng, Gongbin Lan, Guo Long, Honghui Yang, Yajing Xu, Ai Fu, Huimin Yi, Qiquan Wan

**Affiliations:** ^1^ Department of Laboratory Medicine, The Third Xiangya Hospital, Central South University, Changsha, China; ^2^ Xiangya School of Medicine, Central South University, Changsha, China; ^3^ Department of Transplant Surgery, The Second Xiangya Hospital, Central South University, Changsha, China; ^4^ Department of Respiratory and Critical Care Medicine, The Third Xiangya Hospital, Central South University, Changsha, China; ^5^ Department of Hematology, Xiangya Hospital, Central South University, Changsha, China; ^6^ Department of Tuberculosis, The Affiliated Changsha Central Hospital, Hengyang Medical School, University of South China, Changsha, China; ^7^ Department of Surgical Intensive Care Unit, The Third Affiliated Hospital of Sun Yat-sen University, Guangzhou, Guangdong, China; ^8^ Department of Transplant Surgery, The Third Xiangya Hospital, Central South University, Changsha, China; ^9^ Engineering and Technology Research Center for Transplantation Medicine of National Health Comission, the Third Xiangya Hospital, Central South University, Changsha, China

**Keywords:** mucormycosis, *Lichtheimia* species, metagenomic next-generation sequencing, infection, colonization

## Abstract

**Background:**

Mucormycosis is considered the fourth most common invasive fungal disease after candidiasis, aspergillosis and cryptococcosis. *Lichtheimia* species accounted for 5%-29% of all mucormycosis. However, available data on species-specific analysis of *Lichtheimia* infections are limited.

**Methods:**

This study included nine patients hospitalized in five hospitals in two cities in south China with mucormycosis or colonization caused by Lichtheimia species, diagnosed mainly by metagenomic next-generation sequencing (mNGS). The corresponding medical records were reviewed, and the clinical data analyzed included demographic characteristics, site of infection, host factors and type of underlying disease, diagnosis, clinical course, management, and prognosis.

**Results:**

In this study, nine patients with *Lichtheimia* infections or colonization had a recent history of haematological malignancy (33.3%), solid organ transplants (33.3%), pulmonary disease (22.2%), and trauma (11.1%) and were categorized as 11.1% (one case) proven, 66.7% (six cases) probable mucormycosis and 22.2% (two cases) colonization. Pulmonary mucormycosis or colonization was the predominant presentation in 77.8% of cases and mucormycosis caused by *Lichtheimia* resulted in death in four out of seven patients (57.1%).

**Conclusion:**

These cases highlight the importance of early diagnosis and combined therapy for these sporadic yet life-threatening infections. Further studies on the diagnosis and control of *Lichtheimia* infection in China are required.

## Introduction

Mucormycosis has emerged as an increasingly important life-threatening infection in patients with impaired immune system, in diabetic patients with ketoacidosis, and in immunocompetent patients after trauma exposure to contaminated soil (Garcia-Hermoso et al., 2009). Mucormycosis, characterized by rapid local spread, angio-invasion, and tissue necrosis, is an opportunistic infection caused by ubiquitous filamentous fungi of the order Mucorales, showing a 40%-80% mortality rate (Montano and Voigt, 2020). Among the Mucorales, members of the genera *Rhizopus*, *Lichtheimia* and *Mucor* occupy 70%-80% of all causative pathogens (Prakash et al., 2019).

In a global survey and a study in the United States, *Lichtheimia* species accounted for 5% of all mucormycoses (Roden et al., 2005; Alvarez et al., 2009). In contrast, in some studies in Europe, *Lichtheimia* species were identified as the second leading pathogens of mucormycosis, causing 19%-29% of cases (Skiada et al., 2011). *Lichtheimia* infection occurs mainly in Europe (68.2%), followed by Asia (16%) and Africa (9%) (Pan et al., 2020). The most common underlying condition for *Lichtheimia* infection was hematological malignancy (36.3%), followed by trauma/major surgery (27.3%). The sites of *Lichtheimia* infection were mostly skin and soft tissues (45.5%) and lungs (25%) (Pan et al., 2020).

The taxonomy of these genera has undergone profound changes in the last decades: The genus *Lichtheimia* was separated from *Absidia* and *Lichtheimia ramosa* was recognized as a discrete species apart from *Lichtheimia corymbifera* (Hoffmann et al., 2007; Alastruey-Izquierdo et al., 2010). In clinical settings, *L. corymbifera* and *L. ramosa* are the most common pathogens among *Lichtheimia* species. Only four proven cases of mucormycosis caused by *Lichtheimia ornata* have been reported (Legrand et al., 2016; Frealle et al., 2018; Pan et al., 2020). Available data on species-specific analysis of *Lichtheimia* infections are limited. Herein, we described the epidemiologic, pathologic and clinical features of nine cases of mucormycosis or colonization caused by *Lichtheimia* species from five hospitals in south China, which, to our knowledge, represents the largest series of patients with *Lichtheimia* infection or colonization identified by metagenomic next-generation sequencing (mNGS) after 2009 when species identification was supported by molecular data.

## Materials and methods

### Study population

This study included nine patients hospitalized in five hospitals in two cities in south China with mucormycosis or colonization caused by *Lichtheimia* species. The investigations described in this report adhered to the ethical principles of medical research of the Helsinki Declaration. The corresponding medical records were reviewed, and the clinical data analyzed included demographic characteristics, site of infection, host factors and type of underlying disease, diagnosis, clinical course, management, and prognosis.

### Definition

We applied the criteria of the European Organization for Research and Treatment of Cancer and Mycoses Study Group (EORTC/MSG) for proven, probable or possible invasive fungal disease (Donnelly et al., 2020). As such, classifying a case as a proven invasive fungal disease requires histopathologic, cytopathologic, or direct microscopic examination of a specimen obtained by needle aspiration or biopsy in which hyphae are seen and there is evidence of associated tissue damage. Alternatively, a proven case can be described upon detection of a mold by culture in a specimen obtained by aseptic procedures from a normally sterile, clinically or radiologically abnormal site consistent with an infectious disease process. Probable invasive fungal disease is defined as the presence of at least one host factor, a clinical feature and mycologic evidence. A case that meets the criteria for a host factor and a clinical feature, but for which mycological evidence has not been found is considered to have the possible invasive fungal disease. Fungal colonization requires positive mycological cultures or amplification of fungal DNA without signs of infection (Schaal et al., 2015; Donnelly et al., 2020).

### Sampling, culture, histopathology, strain identification, and criteria for proving invasive fungal disease

The collected lung aspirates, blood, nasal secretions, and biopsy tissue samples were processed according to standard protocols and inoculated directly onto Sabouraud dextrose agar (Jiangmen Kailin Trading Co., Ltd, Guangdong, China) at 28 °C for fungal culture. Microscopic morphology was performed by a small steel ring culture method, stained with lactophenol cotton blue or fungal fluorescent staining solution (Baso Medical Device Co., Ltd, Zhuhai, China). Morphological pictures were observed under an Olympus IX71 fluorescence microscope (Japan). Antifungal susceptibility test was performed with the YEASTONE testing kit (Thermo Fisher Scientific, America) using a commercial broth microdilution method according to the manufacturer’s instructions. Left nasal tissue from Case 1 was sent to the pathology laboratory for histological sectioning. The samples were stained with hematoxylin-eosin, Periodic acid-Schiff and Gomori methenamine silver staining and observed under a light microscope (Olympus BX53, Japan).

The obtained isolates were identified through the examination of micro- and macro-morphologic features in accordance with standard morphological criteria (Chakrabarti et al., 2006). Molecular identification of the isolates was performed by comparing the internal transcribed spacer region (ITS) 1-5.8S-ITS4 rDNA gene cluster, partial EF1α and β-actin gene sequences data of the isolated strains with the reference strain data deposited in GenBank.

### mNGS detection

Collected blood, cerebrospinal fluid (CSF), bronchoalveolar lavage fluid (BALF), pus on the skin surface, and stool samples were subjected to mNGS detection (Genskey Co., Ltd, Beijing, China). A total volume of 8 mL of blood, 5 mL of CSF and 5 mL of bloody stool samples were collected for viral and microbial analysis using mNGS. Blood samples were stored at room temperature, and CSF and stool samples were stored at 2-8 °C. The viscous stool samples were homogenized with normal saline. Blood samples and CSF were centrifuged at 1,600 rpm, and stool samples were centrifuged at 12,000 rpm for 10 min at 4 °C to eliminate debris. After centrifugation, 600 uL of supernatant was taken, and DNA was extracted using a micro-sample genomic DNA extraction kit (1901, Genskey, Tianjin). The DNA libraries were constructed by DNA enzyme digestion (200-300 bp), end-repair, poly(A)-tailing, adapter ligation, and polymerase chain reaction (PCR) amplification using an mNGS library construction kit (2012B, Genskey, Tianjin). The quality of the DNA libraries was assessed using an Agilent 2100 Bioanalyzer (Agilent Technologies, Santa Clara, USA) combined with qPCR to measure the adapters before sequencing.

DNA nanospheres were obtained by quantitative addition of 2-3 sets of single-stranded circular DNA, loaded onto sequencing chips and sequenced using the MGISEQ-2000 sequencing platform (MGI, Shenzhen, China). The primary sequencing output was demultiplexed with bcl2fastq v2.20.0.422 with default parameters. Reads were quality-trimmed and low-complexity sequences, and adapter removal was performed with fastp v0.19.5 and Komplexity v0.3.6. Reads mapped to human reference assembly GRCh38 were removed with bowtie2 version 2.3.4.3. The remaining reads were considered as the possible microbial origin. All putative microbial reads were aligned to the company’s microorganism database with SNAP v1.0beta.18. The mapped reads were classified based on the NCBI Taxonomy of the reference genomes.

All species included in the curated pathogen reference databases were collected from books, such as the Manual of Clinical Microbiology, Diagnosis and Illustration of Clinical Microbiology, and NCBI RefSeq genome database. Strictly considered, only one typical high-quality representative strain of each species was selected, whose genome sequence was downloaded from the NCBI RefSeq genome database or NCBI GenBank genome database. Currently, the company’s curated database contains 12,895 bacterial genomes or scaffolds, 11,120 whole genome sequences of viral taxa, 1,582 whole genome sequences of fungal taxa, 312 whole genome sequences of parasites,184 mycoplasma, and 177 mycobacterium.

## Results

The median age of the patients discussed in this report was 51 years (range 45-56 years), of whom 55.6% were male and 44.4% were female. The cases were categorized as 11.1% (one case) proven, 66.7% (six cases) probable mucormycosis and 22.2% (two cases) colonization. Patients included had a recent history of haematological malignancy (33.3%), solid organ transplant (SOT) (33.3%), pulmonary disease (22.2%), and trauma (11.1%). The mucormycosis or colonization most often (77.8%) affected the lungs. *L. ramose* was the most frequently recovered agent identified by mNGS (7/9; 77.8%), followed by *L. corymbifera* (2/9; 22.2%), without any *L. ornate* being detected. All seven patients diagnosed with proven or probable mucormycosis in this study received specific therapy, including four patients who received amphotericin B colloidal dispersion (ABCD) alone or in combination with posaconazole or itraconazole, and one patient each who received liposomal amphotericin B or amphotericin B. In the remaining patient, multiple debridements of the left lower extremity and autologous skin transplantation were sufficient to control the mucormycosis and the recovery was favorable without systemic antifungal therapy. Eventually, three patients survived the *Lichtheimia* infection, four died including 3 of them due to uncontrolled *Lichtheimia* infection and the remaining one died of a cause of unknown. Two patients with asymptomatic airway colonization by *Lichtheimia* diagnosed by mNGS of BALF did not receive specific antifungal therapy and did not develop mucormycosis during a follow-up of over 10 months. **Table 1** showed the demographic characteristics, sites of infection, host factors, types of underlying diseases, management, and prognosis of these nine cases with *Lichtheimia* infections or colonization.

**Table 1:** Characteristics of the 9 cases with infection or colonization caused by *Lichtheimia* species.

	Age/Gender	Underlying disease	Host factor	Clinical presentation	Etiologic agent/ Probability of *Lichtheimia* infection	Antifungal therapy	Outcome
1	52/M	LT	Immunosuppressant	Pulmonary; Cutaneous; Intestinal	*L. ramosa/* proven	ABCD; TTC; posaconazole	Death
2	44/F	LT	Immunosuppressant	Rhinocerebral	*L.ramosa/* probable	ABCD; Posacona-zole amphotericin B (intrathecal)	Death
3	57/F	KT	Immunosuppressant	Pulmonary	*L.corymbifera/* probable	ABCD	Cured
4	46/M	Acute; myelomo-nocytic leukemia; Type 2 diabetes	Neutropenia	Pulmonary	*L. ramosa/* probable	LAMB; posaconazole	Cured
5	49/M	Acute non-lymphocytic leukemia	Neutropenia	Pulmonary	*L. ramosa/* probable	Amphotericin B	Death
6	51/M	Acute leukemia	Neutropenia	Pulmonary	*L. ramosa/* probable	ABCD; Posaconazole	Death
7	56/F	Laceration	Disruption of skin barrier function	Cutaneous	*L. ramose/* probable	None	Cured
8	81/M	Silicosis COPD	Alympho-cytosis	Pulmonary	*L.corymbifera/* colonization	None	Cured
9	32/F	Pulmonary tuberculosis	None	Pulmonary	*L. ramosa/* colonization	None	Cured

LT, liver transplantation; KT, kidney transplantation; ITC, Itraconazole; COPD, chronic obstructive pulmonary disease; ABCD, amphotericin B colloidal dispersion; LAMB, liposomal amphotericin B.

### Proven cases (Case 1)

A 52-year-old male was admitted to the Third Xiangya Hospital of Central South University, Changsha, China, on February 25th, 2022. He was diagnosed with ‘‘hepatitis B virus-related cirrhosis and chronic plus acute hepatic failure with stage II hepatic encephalopathy’’ and underwent a liver transplant (LT) on the same day. The recipient developed acute respiratory failure after LT, requiring ventilator support until day 9 post-LT, and also had acute tubular necrosis requiring intermittent blood purification therapy. He also suffered bleeding from his left nasal septum deviation when the gastric tube was inserted during the operation. The recipient was given basiliximab together with methylprednisolone intravenously for immune induction. Tacrolimus and corticosteroids were used initially for immunosuppressive maintenance therapy. Post-LT antimicrobial prophylaxis included meropenem and teicoplanin. No prophylactic antifungal agent was used after LT.

**Figure 1:**
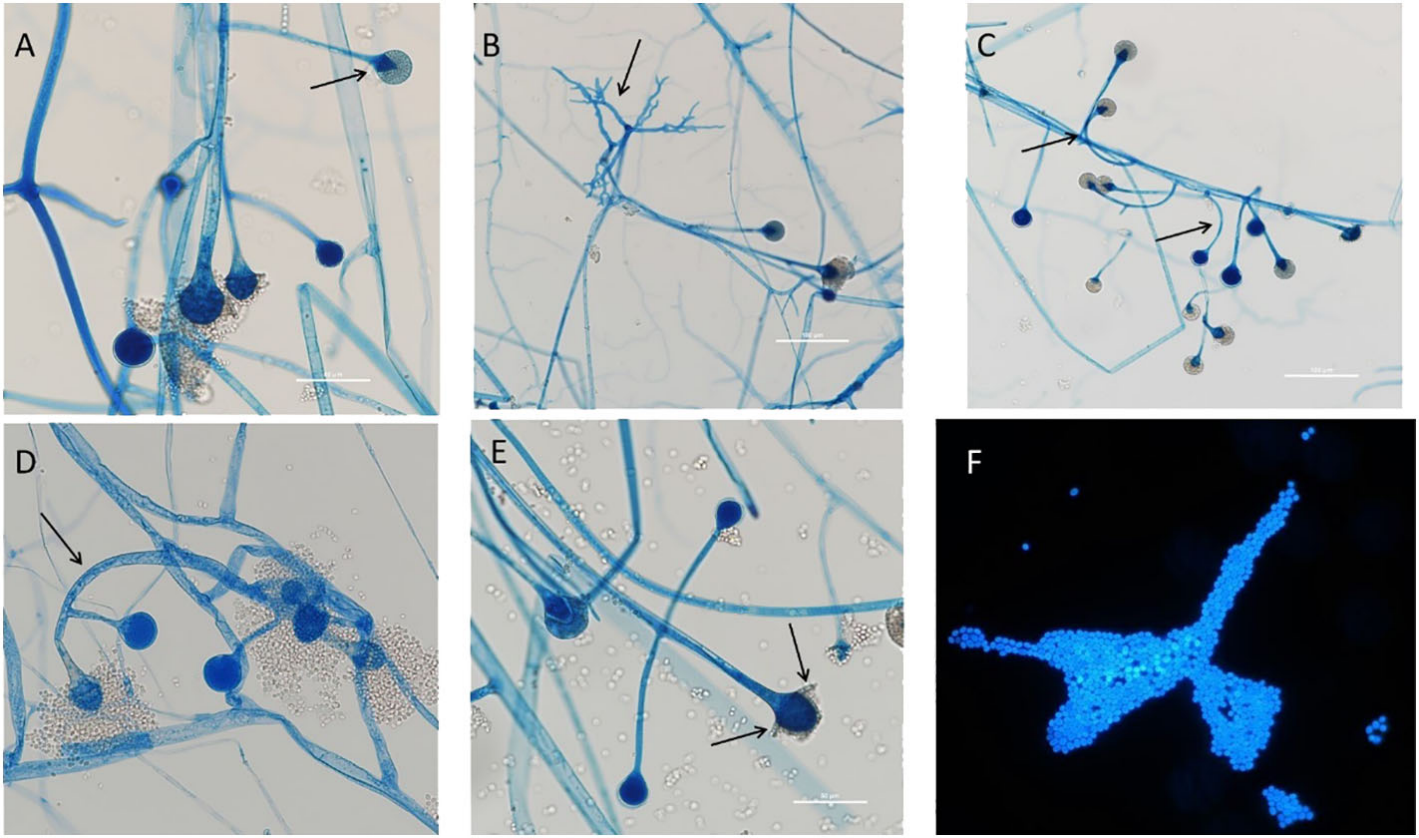
Morphological characteristics of the strain. **(A)** The pear-shaped sporangium and the columella look like comicalness (arrow); **(B)** The rhizoid is not opposite to the sporangiophore (arrow); **(C)** The clustered sporangiophores appear with whorled branches, and some of the side branches are characteristically circinate (arrow); **(D)** The hyphae are smooth, colorless, broad, and nonseptate (arrow, 400×); **(E)** The funnel-formed apophysis (arrow); **(F)** The smooth, oval sporangia spores (1000×).

**Figure 2:**
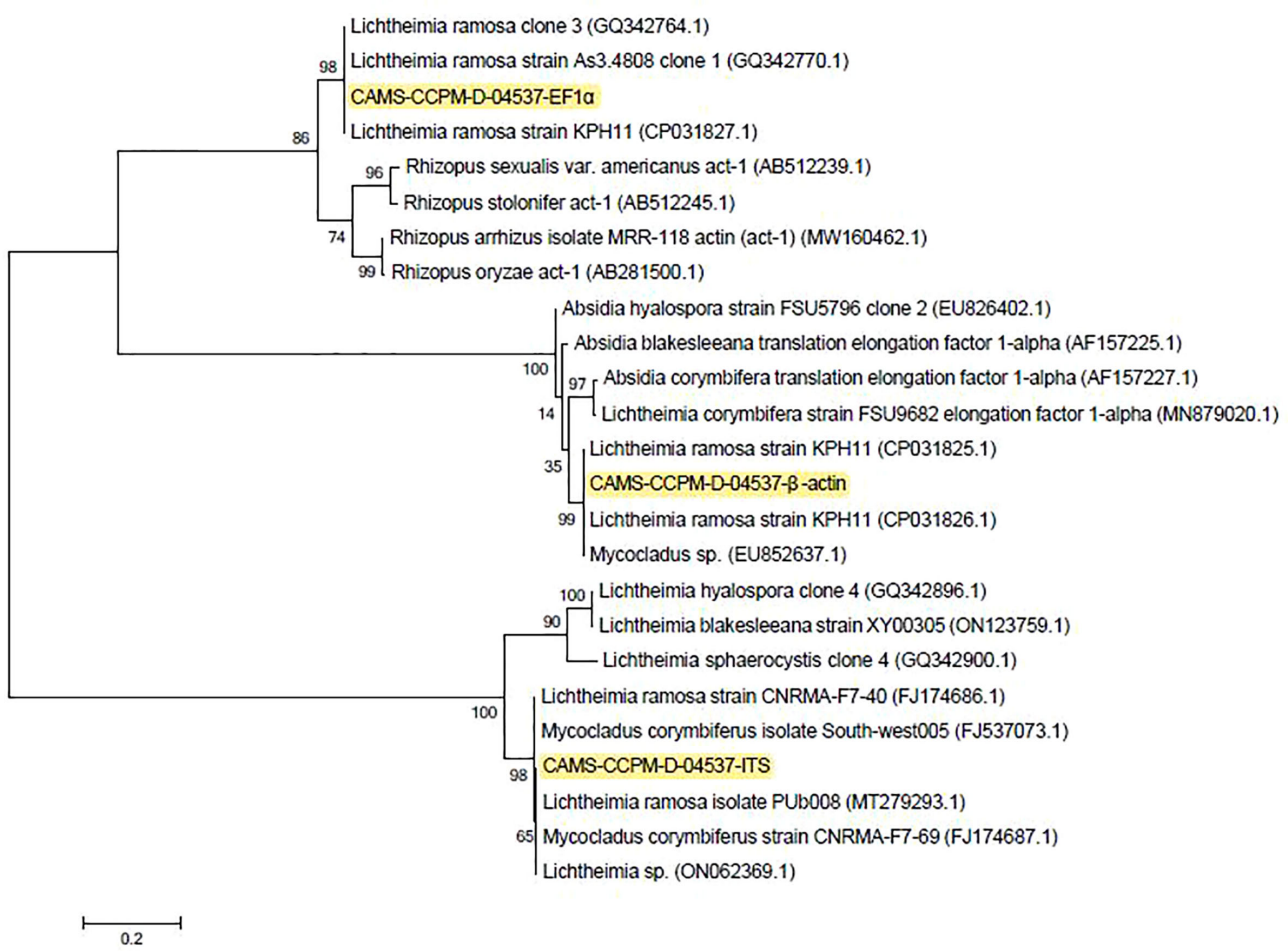
Consensus phylogram (50% majority rule) result from a maximum likelihood (ML) analysis of the internal transcribed spacer region (ITS) alignment, with confidence values bootstrap (BS) and posterior probability (PP) analysis above the branches (>70% for BS from ML analyses, > 0.95 for PP from Bayesian).

The recipient was found to have a ‘‘black nose’’ on day 8 post-LT. Laboratory results revealed a white blood cell count of 7,080/μL. The patient’s immune function was low, with total T cells of 276/μL, CD4-positive T cells of 51/μL, and CD8-positive T cells of 10/μL. The left nasal biopsy tissues were subjected to histopathological examination which later revealed a fungal infection. Meantime, left nasal necrotic tissue, lung aspiration, and left nasal secretions were inoculated beside the bed for fungal culture, and later reported to be positive for Mucorales. An antifungal susceptibility test indicated that the strain was susceptible to amphotericin B, itraconazole, and posaconazole. The morphologic characteristics of the Mucorales were shown in **Figure 1**. Molecular identification of both isolates from lung aspirates and left nasal secretions on Sabouraud dextrose agar was performed *via* the ITS1-5.8S-ITS4 rDNA gene cluster, partial EF1α and β-actin genes sequences showing sequence identity between the two strains. One of them has been deposited in the reference collection of the Branch of Medical Fungi, Center for Pathogen (Virus) Species Preservation, Chinese Academy of Medical Sciences, with an accession number of CAMS-CCPM-D-04537. The ITS1-5.8S-ITS4 rDNA gene cluster, partial EF1α and β-actin genes of CAMS-CCPM-D-04537 have been deposited in GenBank (accession numbers ON248968, ON637261, and ON620270, respectively). Phylogenetic analysis inferred the strain to be *L. ramosa* (**Figure 2**).

The recipient underwent three mNGS detection using blood samples on day 8, 14, and 20 post-LT, with *L. ramosa* specific reads of 2174, 41 and 0. Computed tomography (CT) scans (on day 10 and 18 post-LT) showed pansinusitis and scattered nodulars in the lungs, in some of which small thickly walled cavities were found, accompanied by bilateral pleural effusion. Tacrolimus was discontinued on day 8 post-LT, and only a very low dosage of steroid (5-10 mg daily) was reserved. mNGS detection of CSF was also performed on day 13 post-LT and the results were negative.

The recipient developed severe lower intestinal bleeding on day 15 post-LT and underwent gelatin-sponge embolization of a branch of the jejunal artery on the same day. mNGS detection of bloody stool was performed on day 24 post-LT, showing not only *L. ramosa*, but also *L. corymbifera*. Repeated blood and ascites cultures for bacteria and fungi were negative. Repeated sputum cultures from day 10 onwards after LT were Mucorales. All sputum cultures grew *Acinetobacter baumannii* since day 20 post-LT. A combination of posaconazole or itraconazole and ABCD was used to treat the multiple-sites *L. ramosa* infection according to mycologic results. Despite aggressive blood transfusion, hemostasis, and anti-infective treatment, the patient’s condition deteriorated and hemorrhagic shock developed on day 26 post-LT. His family refused to perform exploratory laparotomy, and the patient was discharged the same day.

### Probable cases (Case 2-7)

The median age of the six patients with probable *Lichtheimia* infection was 50 years (range 45.5-56.3 years), of whom three were males and three females. Haematological malignancy and SOT were reported in three and two patients, respectively, while Case 7 had trauma. Pulmonary mucormycosis was the predominant presentation as it was noted in 66.7% of cases.

#### Case 2

**Figure 3:**
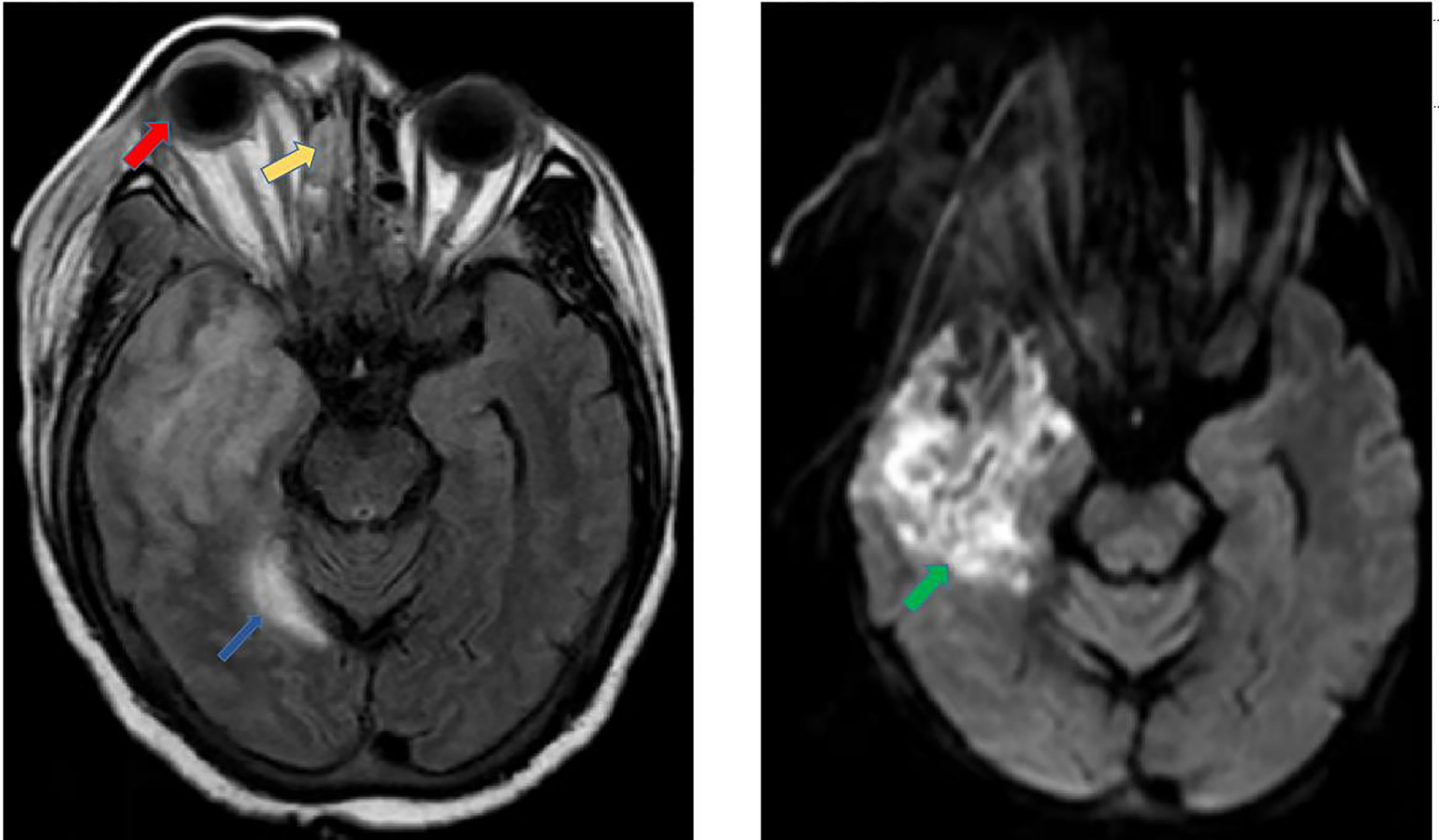
Enhanced magnetic resonance imaging and diffusion weighted imaging. The MRI results show that multiple recent infarcts in the right frontal and temporal regions (green arrow), protrusion of the right eyeball (red arrow), ethmoid sinusitis (yellow arrow), and a few paratentorial subdural/epidural hematoma (blue arrow).

A 44-year-old Chinese female was admitted to the Third Affiliated Hospital of Sun Yat-sen University, Guangzhou, China, on December 29th, 2021. Physical examination on admission revealed swelling in both eyes, especially in the right eye, and conjunctival congestion in the right eye. She had been diagnosed with ‘‘biliary cirrhosis secondary to intrahepatic cholangiolithiasis and chronic plus acute hepatic failure with stage III hepatic encephalopathy’’ and underwent LT of graft from donation after citizen’s death donor on the same day. Caspofungin was used as prophylaxis for antifungal therapy immediately after LT. Postoperative physical examination in the intensive care unit found that the swelling of the patient’s right eye was more severe than before, and that the left nasal cavity was bleeding. A gelatine sponge was given to stop the bleeding. Enhanced magnetic resonance imaging and diffusion weighted imaging of the head on day 4 post-LT showed: 1. Multiple recent infarcts in the right frontal and temporal; 2. Protrusion of the right eyeball; 3. Pansinusitis; and 4. A few paratentorial subdural/epidural hematoma (**Figure 3**). The recipient underwent mNGS detection using CSF on day 7 post-LT, with *L. ramosa* specific reads of 2581. Caspofungin was replaced by ABCD (intravenous) and posaconazole (oral) as well as amphotericin B (intrathecal) to treat the *L. ramosa* infection. The patient’s condition deteriorated with persistent fever and lethargy, eye swelling, ulcer around her right eye, and continued deterioration of liver function. Her family refused to continue treatment, and she was discharged on day 8 post-LT.

#### Case 3

**Figure 4:**
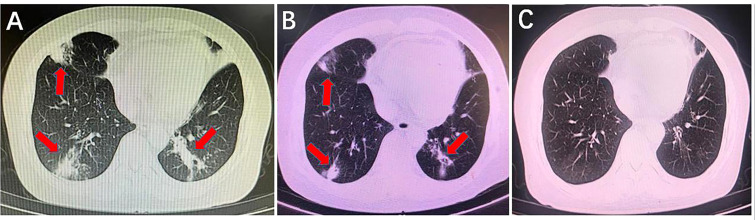
Comparison of lung computed tomography scan results before and after using anti-mucormycotic drugs. **(A)** Before using anti-mucormycotic drugs, there were scattered bands and patches with increased density in the anterior segment of the right upper lobe, the lingual segment of the left upper lobe, the middle lobe of the right lung and the lower lobe of both lungs (red arrow). **(B)** There was a slight decrease in infectious exudation of both lungs after the combination of amphotericin B colloidal dispersion and posaconazole was used for 13 days (red arrow). **(C)** After more than 2 months of posaconazole follow-up, infectious lesions of both lungs completely disappeared.

A 57-year-old Chinese female was admitted to the Second Xiangya Hospital, Central South University, Changsha, China, on January 22th, 2022, because of cough with shortness of breath for 2 days, 5 years after renal transplantation for chronic renal insufficiency due to chronic nephritis. On admission, physical examination showed clear respiratory sounds in both lungs, and no rales were heard. She was diagnosed with ‘‘pulmonary infection and kidney transplantation’’. After admission, the dosage of mycophenolate mofetil was reduced. The patient was given piperacillin/tazobactam and caspofungin for anti-infection for 4 days. However, the patient’s cough worsened, and the CT examination showed a significant increase in infectious exudation in both lungs (**Figure 4A**). Fibrobronchoscopy was performed, and BALF was retained for mNGS to identify the pathogen, which showed *L. corymbifera* infection with specific reads of 13. Since then, caspofungin was discontinued. After 13 days of combined treatment with ABCD and posaconazole, the patient’s cough improved significantly, and the CT scan showed a significant reduction in lung lesions (**Figure 4B**). Posaconazole alone was then used for 3 months until the CT scan demonstrated that the *L. corymbifera* infection was resolved (**Figure 4C**).

#### Case 4

A 46-year-old man, diagnosed with ‘‘acute myelomonocytic leukemia; type 2 diabetes; pneumonia’’, was admitted to the Second Xiangya Hospital of Central South University, Changsha, China, on October 26th, 2020. Physical examination on admission showed no abnormality. On the fourth day after admission, idarubicin, etoposide and cytarabine were given to the patient for the first chemotherapy. Piperacillin/tazobactam was prescribed to the patient as a prophylactive antibiotic during and after the chemotherapy. Prophylactic antifungal drugs were not used. On the ninth day after admission, the patient developed fever, cough, and purulent sputum, with a maximum temperature of 38.5 °C. Laboratory tests showed that the patient had neutropenia with a neutrophil count of 90/μL and the neutropenia lasted for 10 days. Piperacillin/tazobactam was replaced by meropenem, teicotranin and posaconazole as anti-infection agents. CT scan on the 16th day of admission showed scattered exudation and nodules in both lungs. Posaconazole was replaced by a combination of caspofungin and voriconazole, but the patient’s temperature was poorly controlled. CT scan on the 28th day of admission demonstrated multiple consolidations in both lungs with bilateral pleural effusion. On the 30th day after admission, caspofungin was replaced by liposomal amphotericin B. On the 32nd day after admission, the patient underwent bronchofibroscopy, which revealed a gray plaque on the left bronchus medially. *L. ramosa* and *Aspergillus fumigatus* were detected by mNGS in BALF with specific reads of 41 and 15, respectively. The patient was treated with liposomal amphotericin B for 2 weeks, followed by posaconazole, because of the mixed pulmonary infection caused by *L. ramosa* and *A. fumigatus*, and then he was discharged in good condition.

#### Case 5

A 49-year-old man, diagnosed with ‘‘acute non-lymphocytic leukemia’’, was admitted to the Xiangya Hospital of Central South University, Changsha, China, on March 2nd, 2022. Upon physical examination at admission, no abnormality was revealed. CT scan on the first day after admission demonstrated multiple mixed ground glass nodules in both lungs. Posaconazole had been used for 28 days from the third day of his admission. On the ninth day after admission, idarubicin and cytarabine were given to the patient for the first chemotherapy. After chemotherapy, the patient developed neutropenia and fever, and blood culture showed *Escherichia coli* infection. After administration of meropenem, the infection was controlled. Voriconazole had been used for 18 days from the thirty-first day of his admission. On the 43rd day after admission, venecra and decitabine were given for the second chemotherapy. On the fifth day of chemotherapy, granulocytosis and fever recurred with increased procalcitonin. CT scan showed new clumps and cords of increased density in the right upper apical lung segment and the posterior basal segment of both lower lungs. The patient was prescribed amphotericin B from the forty-eighth day of his admission. On the 52nd day after admission, mNGS detection of the blood was performed and later revealed *L. ramosa* infection with specific reads of 2579. The patient was discharged on the 67th day after admission and transferred to a local hospital for further treatment. Telephone follow-up revealed that the patient died after the transfer but the family refused to give details.

#### Case 6

A 51-year-old man, diagnosed with ‘‘acute leukemia; pulmonary infection with bilateral pleural effusion’’, was admitted to the Xiangya Hospital of Central South University, Changsha, China, on April 18th, 2022. On the fourth day after admission, the patient was given the first chemotherapy with azacitidine and venecra. During the chemotherapy, the patient developed fever and cough and expectoration. The blood cultures showed *A. baumannii* infection. Meropenem, piperacillin/tazobactam and tigecycline were given as anti-infection drugs, and the patient’s temperature was controlled. On the 23rd day of admission, low fever reappeared. Sputum culture detected *A. baumannii*. Piperacillin/tazobactam and tigecycline were given to treat the infection. On the 26th day of admission, the CT scan showed patchy high-density shadows in the right upper apex and middle lung. Subpleural patches of increased density and consolidation in the lower lobe of both lungs. On the 31st day of admission, the patient developed dyspnea with hypoxia and hypotension. mNGS of blood detected *L. ramosa* with specific reads of 295. Voriconazole had been used for 31 days before the mNGS. The patient had neutropenia from the time of admission until the onset of the *L. ramosa* infection. Posaconazole and ABCD were immediately added to the patient’s treatment. The patient’s temperature dropped to normal, but the dyspnea gradually worsened. Endotracheal intubation and ventilator support were performed. Two days later, the patient was in a coma and his blood pressure could not be maintained. The patient’s family refused to continue treatment, and he was discharged on the 33rd day after admission.

#### Case 7

A 56-year-old female diagnosed with ‘‘laceration, necrosis and infection of the left lower extremity due to a traffic accident’’, was admitted to the Third Xiangya Hospital of Central South University, Changsha, China, on September 7th, 2020. No prophylactic antifungal agents were used after her traffic accident. Physical examination on admission showed necrosis of the skin of the left lower extremity with yellow-white pus outflow and fishy odor, and pale skin of the left lower extremity. mNGS detection of the pus from the skin surface of the left lower extremity was performed on the day of admission and later showed the detection of *L. ramosa*, with specific reads of 8. After admission, the patient underwent multiple debridements of the left lower extremity and autologous skin transplantation, and was given cefuroxime and other antibiotics for anti-infection treatment. The patient was discharged on the 57th day after admission in good condition with a largely healed wound. She received no amphotericin B and was healed by debridement alone.

### Colonization case (Case 8, 9)

#### Case 8

A 81-year-old man, diagnosed with ‘‘chronic obstructive pulmonary disease with pneumonia secondary to silicosis’’, was admitted to the Third Xiangya Hospital of Central South University, Changsha, China, on September 26th, 2021. Physical examination on admission revealed a barrel chest with low breath sounds and a small number of dry rales in both lower lungs. CT scan was done on the admission day and showed features of chronic bronchial disease, emphysema, multiple pulmonary bullae formation in both lungs, and pleomorphic lesions of both lungs, which were considered to be the result of silicosis. On the sixth day after admission, a bronchoscopy was performed. mNGS examination of BALF showed *Klebsiella pneumoniae*, *Candida albicans*, and *L. corymbifera* with specific reads of 183, 161 and 7, respectively. None of antifungal agent was used before the mNGS examination. The patient was given piperacillin/tazobactam for 6 days to fight the infection and then meropenem for another 5 days. No antifungal drugs were used during hospitalization. The patient’s condition did not improve or worsen significantly. After 11 days in the hospital, he was transferred to a local hospital for further treatment due to financial difficulties. During a follow-up of over 10 months, the patient did not develop any signs of *Lichtheimia* infection.

#### Case 9

A 31-year-old female, diagnosed with ‘‘pulmonary tuberculosis of the left upper lung’’, was admitted to the Changsha Central Hospital, Changsha, China, on January 22nd, 2021. Physical examination at admission showed no obvious abnormality. After admission, CT scan showed multiple patchy and speckled shadows with increased density, and blurred margins in the posterior apical segment of the left upper lobe. The patient was given ethambutol, pyrazinamide, rifampicin, and isoniazid to fight tuberculosis. Five days after admission, mNGS of BALF showed *Pseudomonas aeruginosa*, *Streptococcus parasanguinis*, *Aspergillus glaucus*, and *L. ramosa* with specific reads of 98, 199, 6, and 3, respectively. None of antifungal agent was used before the mNGS examination. Interferon gamma release assay showed positive, whereas multiple sputum smears were negative for acid-fast bacilli. The family refused to continue diagnostic antituberculous therapy. As a result, the patient was discharged on the 10th day after admission and transferred to a higher hospital for further treatment. During a follow-up of over 19 months, the *Lichtheimia* colonization did not developmucormycosis.

## Discussion

Although mucormycosis remains a relatively uncommon disease compared to candidiasis and aspergillosis, much attention has been focused on it because of its aggressive clinical course and high mortality rate, even with appropriate medical management (Wingard, 2006). In contrast to *Rhizopus*, the most common agent of mucormycosis in diabetic patients, *Lichtheimia* infections were primarily associated with hematological malignancies, SOT, prolonged severe neutropenia, and major skin barrier damage, in line with our present study, where all cases of *Lichtheimia* infection had recent haematological malignancy, SOT or skin barrier damage (Schwartze and Jacobsen, 2014; Pan et al., 2020).

The clinical forms of mucormycosis include rhinocerebral, pulmonary, cutaneous, disseminated, and gastrointestinal forms. In immunocompetent hosts, cutaneous mucormycosis is common following trauma. The rhino-cerebral form of mucormycosis is most commonly seen in patients with diabetes mellitus, whereas pulmonary mucormycosis in patients with haematological malignancy and transplant recipients (Prakash and Chakrabarti, 2019).

A common clinical manifestation caused by *Lichtheimia* species is cutaneous and subcutaneous infections. For example, *L. corymbifera* was often found in skin lesions (Pan et al., 2020). These cases are generally associated with previous wounds or fractures due to traumatic accidents or surgery (Schwartze and Jacobsen, 2014). Outbreaks of *Lichtheimia* species are transmitted by direct contact. Several outbreaks of *Lichtheimia* infections have been reported and the supposed sources include bandages, linen, traumatic inoculation, and person-to-person transmission (Llata et al., 2011; Poirier et al., 2013; Cheng et al., 2016; Frealle et al., 2018). Fréalle E et al. reported that *L. ramosa* and *L. ornata* were present in five and two cases in burn patients with mucormycosis, respectively (Frealle et al., 2018). Actually, in our present study, although pulmonary mucormycosis was the predominant presentation, there were two cases of rhinorrhea during intraoperative insertion of a gastric tube and one case of laceration before the *Lichtheimia* infection.

Pulmonary infections with *L. corymbifera* have also been reported in patients with different underlying diseases, including bone marrow and SOT, uncontrolled diabetes, and leukaemia (Borras et al., 2010; Dabritz et al., 2011; Kleinotiene et al., 2013; Zaki et al., 2014; Pan et al., 2020). CT scan of pulmonary mucormycosis mainly showed infiltration or consolidation of lungs, thick-walled cavities and pleural effusion. Inhalation of asexual spores (sporangiospores) is believed to be the main route of infection with mucormycetes; and thus, the infection commonly manifests in the respiratory tract (Lanternier et al., 2012). In our present study, pulmonary mucormycosis or colonization was the predominant presentation, as it was noted in 77.8% of our cases, a result that agrees with the literature.

*Lichtheimia* infection is a rare but emerging fungal infection with high mortality. *L. corymbifera*, *L. ramosa* and *L. ornata* are clinically relevant members of the genus *Lichtheimia* (Alastruey-Izquierdo et al., 2010). Although the pathogenic potential of both *L. corymbifera* and *L. ramosa* is well documented in human cases, only one clinical isolate of *L. ornata* and four proven cases with mucormycosis caused by *L. ornata* have been described (Alastruey-Izquierdo et al., 2010; Legrand et al., 2016; Frealle et al., 2018; Pan et al., 2020). The mortality rate due to *L. ramosa* infection (42.9%) was higher than that of *L. corymbifera* (27.3%), similar to our present result that *L. ramosa* infection led to a higher mortality rate of 66.7% (4/6) than that of *L. corymbifera* infection (0/1; 0%) (Pan et al., 2020).

To date, four cases of mucormycosis caused by *Lichtheimia* species have been reported in LT recipients worldwide (Viscoli et al., 1997; Woo et al., 2010; Zaki et al., 2014). Here, we reported nine cases of infection or colonization caused by *Lichtheimia* species, including two LT cases of *Lichtheimia* infection. According to the EORTC/MSG criteria, Case 1 was diagnosed as a proven mucormycosis caused by *L. ramosa* involving multiple organs, including the lung, nose, and intestine. A co-infection by *L. corymbifera* was also considered to be present in the intestine. The infection most likely originated from colonization of *L. ramosa* in the pre-transplant sinuses because the CT scan showed inflammation in all paranasal sinuses, and he was bleeding from his left nose at the time of gastric tube insertion. *L. ramosa* was isolated from nasal necrotic tissue, lung aspirates and left nasal secretions by culture and detected from stool sample by mNGS, so we hypothesized that the route of the infection was through inhalation and ingestion of its spores from the nose to the lungs and intestine.

Infections caused by *Lichtheimia* species were considered to be more common in Europe and relatively rare in other regions (Prakash et al., 2019). But the true incidence/prevalence of mucormycosis may be higher, as many of the cases remain undiagnosed due to the difficulty in collecting samples from deep tissue and the low sensitivity of diagnostic tests. Mucormycosis has also been sporadically reported in China, but in most cases, no further DNA sequencing has been performed to identify the species of mucormycetes. Therefore, we postulate that infections caused by *Lichtheimia* species were grossly underestimated in China.

Among the nine recorded cases, the lungs were the major site of infection or colonization (7/9, 77.8%), with multisite mucormycosis evident in one of the pulmonary patients, while rhinocerebral and cutaneous involvement each constituted only 11.1%, in line with the Egypt study where all four patients with *Lichtheimia* infection developed pulmonary mucormycosis (Zaki et al., 2014).

The significance of mucosal respiratory colonization, as opposed to an infection, and its optimal management are debatable. In a review of over 630 lung transplant recipients at Stanford University, pulmonary colonization by molds was 1.5-fold more common than invasive mold infection, and only a minority of patients developed invasive mold infection (Vazquez et al., 2015). Our present study was in agreement with the previous study, as neither of the two cases with *Lichtheimia* colonization progressed to mucormycosis.

We searched PubMed for articles reporting the infections caused by of Lichtheimia species after 2009 when species identification was supported by molecular data. During the period 2009-2022, 35 articles containing 80 cases of *Lichtheimia* infection, identified by ITS, PCR and mNGS, were published (Llata et al., 2011; Millon et al., 2016; Frealle et al., 2018; Prakash et al., 2019; Pan et al., 2020; Wand et al., 2020; Liu et al., 2021; Zhang et al., 2021; Colman et al., 2022). Although mNGS is a rapid and non-invasive diagnostic method and its early use is recommended when a rare pathogen-related infection is suspected, especially in immunodeficient individuals requiring urgent treatment, only two cases of *Lichtheimia* infection identified by mNGS were reported (Liu et al., 2021; Zhang et al., 2021; He et al., 2022).

The early symptoms of *Lichtheimia* infections are not typical. Most patients with *Lichtheimia* infections are treated empirically without a definite diagnosis. The traditional etiological detection methods are ineffective, so clinical diagnosis is still faced with great challenges, and the delay of treatment seriously affects the prognosis of patients (Yohai et al., 1994). Therefore, it is crucial to quickly and accurately identify the *Lichtheimia* species, and then optimize medical therapy. Although early diagnosis and monitoring of mucormycosis by detection of circulating DNA in serum using quantitative PCR could obtain the best cost/benefit ratio, we found that mNGS, a new genomics-based pathogen detection technology, is a useful tool for effectively detecting *Lichtheimia* species in a short time (about 24 hours) and promptly initiating the treatment against *Lichtheimia* infection, since in our nine cases with *Lichtheimia* infection or colonization identified by mNGS, only Case 1 has also been confirmed by cultures with the remaining eight patients’ *Lichtheimia* cultures were negative. mNGS can simultaneously detect bacterial, parasitic, viral and the other fungal infections. Most of our cases are severely ill patients with infections. Delayed diagnosis and treatment of any etiological agents impacts the outcome of these patients. So mNGS is a more suitable choice than quantitative PCR in our series of cases. It is an important problem that most of our data are not confirmed by culture. In Case 1, left nasal necrotic tissue, lung aspiration fluid and left nasal secretions were inoculated beside the bed for fungal culture and were later reported all positive for Mucorales. Interestingly, only the specimens inoculated beside the bed for fungal culture showed positive Mucorales culture results, whereas the other specimens inoculated in the microbiology room failed to show Mucorales growth, suggesting that the vitality of Mucorales is very fragile and timely inoculation is crucial to improve the positive rate of Mucorales culture. Liu et al. also reported that CSF specimen was examined using traditional culture and mNGS. Traditional culture produced a negative result, while the mNGS revealed the presence of *L. ramosa* (Liu et al., 2021). Thus, mNGS can be an important auxiliary method to diagnose *Lichtheimia* infections, especially when patients with an active infection have a negative culture result, and it is paramount to improve the clinical cognition of this technology.

Although mNGS has the capability of rapid, sensitive, and accurate pathogen identification, choosing when to perform mNGS is critical. Case 2 underwent mNGS detection using CSF on day 7 post-LT. However, physical examination on admission revealed swelling in both eyes, especially in the right eye, and conjunctival congestion in the right eye. In Case 5, on the 48th day after admission, granulocytosis and fever recurred with increased procalcitonin. However, on the 52nd day after admission, mNGS detection of the blood was performed. In Case 6, on the 23rd day of admission, low fever reappeared. On the 31st day of admission, the patient developed dyspnea with hypoxia and hypotension and mNGS of blood was detected. Although they were treated with appropriate antifungal agents after mNGS all these patients died. However, earlier use of NGS detection can help these patients to be diagnosed early and given appropriate treatment, which may contribute to their better prognosis.

Once the discovery of *Lichtheimia*’s characteristic by culture, biopsy or mNGS, standard medication should be established as soon as possible, so as not to delay the treatment. Amphotericin B and its liposomal form were the drugs of choice for the treatment of mucormycosis (Roden et al., 2005). In addition, the combination of intravenous administration and airway inhalation of amphotericin B for pulmonary mucormycosis and intravenous administration and intrathecal injection of amphotericin B for intracranial mucormycosis may be an effective strategy(Chen et al., 2021). Posaconazole and isavuconazole are commonly used in salvage therapy for mucormycosis. Isavuconazole has been approved as the first-line agent against Mucorales in cases where amphotericin B therapy is not appropriate (Marty et al., 2016). However, some studies have reported that posaconazole and isavuconazole prophylaxis were used in 12%-55% and 17%-30.8% of patients who developed mucormycosis, respectively (Auberger et al., 2012; Cho et al., 2015; Rausch et al., 2018). Meanwhile, the effectiveness of posaconazole is still controversial (Vehreschild et al., 2013). According to our present study and previous studies, besides the three drugs mentioned above, itraconazole is also a drug of choice for the treatment of mucormycosis caused by *Lichtheimia* species (Pan et al., 2020). Aggressive antifungal therapy combined with surgical debridement of affected tissues is guaranteed a better prognosis.

There were several limitations in our study due to its retrospective design and the absence of a control group. Furthermore, our *Lichtheimia* infection or colonization cases came from five different hospitals, so the homogeneity of the study cannot be guaranteed. In addition, pertinent information was missing in many of the cases. However, considering the scarcity of relevant data on the subject, we believe that the current study may aid clinicians in the future management of *Lichtheimia* infection or colonization.

In summary, we presented a report of nine cases of mucormycosis or colonization caused by *Lichtheimia* species in south China. Our cases of mucormycosis or colonization caused by *Lichtheimia* species are unique, with multiple sites involvement, and represent the largest series of patients with *Lichtheimia* infection or colonization identified by mNGS after 2009, when species identifications were supported by molecular data. We also found that in Case 1, mNGS detection of stool sample showed not only *L. ramosa* but also *L. corymbifera*. Therefore, *Lichtheimia* infection may be underestimated. Due to the high mortality rate of previously and currently reported cases of infections caused by *Lichtheimia*, in particular *L. ramose*, more efforts are urgently needed in China to aggressively diagnose and initiate treatment through the use of novel molecular assays, including timely and rational systematic application of antifungal medications and potentially surgical debridement or resection. We believe that an early and aggressive diagnostic approach utilizing novel diagnostic techniques such as mNGS may improve patient outcomes.

## Data availability statement

The datasets presented in this study can be found in online repositories. The names of the repository/repositories and accession number(s) can be found in the article/supplementary material.

## Ethics statement

The studies involving human participants were reviewed and approved by The Institutional Review Board of the Third-Xiangya Hospital of Central South University. The patients/participants provided their written informed consent to participate in this study.

## Author contributions

QW conceived and designed the study. All authors acquired the data. LC and WP analyzed and interpreted the data. QW, LC, and WP drafted the manuscript. All authors reviewed the manuscript and vouch for the accuracy and completeness of the data and for the adherence of the study to the protocol. QW, LC, and WP had full access to all of the data in the study. All authors contributed to the article and approved the submitted version.
